# Prognostic value of plasma galectin‐3 levels after aneurysmal subarachnoid hemorrhage

**DOI:** 10.1002/brb3.543

**Published:** 2016-08-02

**Authors:** Hua Liu, Yong Liu, Jinbing Zhao, Hongyi Liu, Shengxue He

**Affiliations:** ^1^ Department of NeurosurgeryThe Nanjing Brain Hospital Affiliated to Nanjing Medical UniversityNanjingJiangsu ProvinceChina; ^2^ Department of NeurosurgeryThe First People's Hospital of Kunshan Affiliated to Jiangsu UniversitySuzhouJiangsu ProvinceChina

**Keywords:** Galectin‐3, intracranial aneurysmal, prognosis, severity, subarachnoid hemorrhage

## Abstract

**Background:**

Inflammatory responses are correlated with secondary brain injury after aneurysmal subarachnoid hemorrhage (aSAH). Galectin‐3 (Gal‐3) is a novel biomarker reflecting inflammation status, and its elevated circulating levels are associated with poor prognosis of some inflammatory diseases. The aim of this study was to evaluate the relationship between Gal‐3 plasma levels and prognosis in a group of aSAH patients.

**Materials and Methods:**

We assessed plasma Gal‐3 levels in 120 patients and 120 healthy individuals. 6‐month clinical outcomes included mortality and unfavorable outcome (Glasgow Outcome Scale score of 1–3). Associations of plasma Gal‐3 levels with clinical outcomes were investigated using multivariate analysis.

**Results:**

Patients showed significantly higher Gal‐3 levels as compared to controls. Circulating Gal‐3 was significantly and independently associated with 6‐month clinical outcomes in the logistic regression analysis. Moreover, we observed a significant correlation between circulating Gal‐3 and World Federation of Neurological Surgeons scores and modified Fisher scores. Furthermore, Gal‐3 possessed high area under receiver operating characteristic curve for prognostic assessment.

**Conclusion:**

Our findings indicate the associations between Gal‐3 levels and the severity and poor prognosis following aSAH. This suggests the possible role of Gal‐3 in the prognostic prediction after aSAH.

## 1 Introduction

Aneurysmal subarachnoid hemorrhage (aSAH) is a common neurological disease (Rodríguez‐Rodríguez, Egea‐ Guerrero, Ruiz de Azúa‐López, & Murillo‐Cabezas, 2014; de Rooij, Linn, van der Plas, Algra, & Rinkel, 2007; Shen et al., 2015). Hemorrhagic brain injury contributes to poor outcome of aSAH (De Marchis et al., 2015; Fujii et al., 2013; Ji & Chen, 2016; Suwatcharangkoon et al., 2016). Inflammation is an important mechanism underlying hemorrhagic brain injury (Badjatia et al., 2015; Ma, Zhou, Yan, Qu, & Bu, 2015). Some inflammation‐related biomarkers have shown high prognostic value for aSAH (Kim et al., 2008; Pan, Yan, Hassan, Fang, & Chen, 2016; Romero, Bertolini Ede, Figueiredo, & Teixeira, 2012). Galectin‐3 (Gal‐3) belongs to the galectin family and is characterized by a conserved sequence within the carbohydrate recognition domain that has an affinity for β‐galactoside residues (Dumic, Dabelic, & Flögel, 2006; Nangia‐Makker, Nakahara, Hogan, & Raz, 2007; Pricci et al., 2000). It is involved in cell adhesion, proliferation, clearance, apoptosis, cell activation, cell migration, and phagocytosis (Argüeso, Mauris, & Uchino, 2015; Chen, Hou, Zhang, Chen, & He, 2015; Li, Li, & Gao, 2014; de Oliveira et al., 2015). Gla‐3 was recently identified as a proinflammatory protein and correlated with the occurrence and progression of cancer, systemic lupus erythematosus, rheumatoid arthritis, chronic heart failure, and systemic sclerosis (Issa et al., 2015; Koca et al., 2014; Nielsen et al., 2014; Piper, de Courcey, Sherwood, Amin‐Youssef, & McDonagh, 2016; Zeinali, Adelinik, Papian, Khorramdelazad, & Abedinzadeh, 2015). Interestingly, activated glia can express Gal‐3 (Jaquenod De Giusti et al., 2011); moreover, Gal‐3 is required for resident microglia activation and proliferation in response to ischemic injury (Lalancette‐Hébert et al., 2012). Furthermore, circulating Gal‐3 levels had the obvious diagnostic value for mild traumatic brain injury in adults (Shan et al., 2016) and independently predicted the occurrence of postoperative strokes among female subjects who undergo carotid endarterectomy (Edsfeldt et al., 2016). Notably, plasma Gal‐3 levels had high prognostic value for severe traumatic brain injury (Shen et al., 2016). The above‐mentioned accumulating evidence indicates that Gal‐3 might have the potential to identify aSAH patients at risk of poor outcome. The aim of the present study was to investigate the impact of galectin‐3 levels on the severity and prognosis of aSAH patients.

## 2 Materials and Methods

### 2.1 Study population

This prospective study included patients with aSAH who were consecutively admitted to the Department of Neurosurgery, Nanjing Brain Hospital Affiliated to Nanjing Medical University from October 2013 to January 2015. Inclusion criteria were that (1) first‐ever SAH admitted within 24 hr of hemorrhage onset, (2) single intracranial aneurysm confirmed by computerized tomography (CT) angiography with or without digital subtraction angiography, and (3) intracranial aneurysm treated through clipping or coiling within the 48 hr after admission. Exclusion criteria were that (1) <18 years of age, (2) rebleeding after admission, (3) suspected pseudoaneurysm, (4) previous neurological disease such as trauma, intracerebral hemorrhage, cerebral infarction and multiple sclerosis, (5) prior use of anticoagulants or antiaggregant drugs, (6) infection within recent 1 month, and (7) systemic diseases like chronic heart failure, uremia, chronic obstruction pulmonary disease, liver cirrhosis and malignancy. During the period of January 2014 to January 2015, a group of healthy individuals (control group) were enrolled. The protocol was approved by the ethics Committee at the Nanjing Brain Hospital Affiliated to Nanjing Medical University and conducted according to the Helsinki Declaration of 1971, as revised in 1983. Written informed consents were given by the subjects or their relatives.

### 2.2 Assessment

Demographic parameters and baseline characteristics were investigated at admission. The World Federation of Neurological Surgeons (WFNS) scale and modified Fisher grading system were used to assess the clinical and radiological severity of aSAH as described previously (Drake, 1988; Fisher, Kistler, & Davis, 1980). We also recorded hydrocephalus, intraventricular hemorrhage or cerebral infarction in accordance with brain CT scan. Symptomatic cerebral vasospasm was defined based on the previous reports (Claassen et al., 2001; Frontera et al., 2009). Glasgow outcome scale score of 1–3 was considered as an unfavorable outcome. During 6‐month follow‐up, the patients or their relatives were contacted by telephone every 2 weeks. The primary endpoint was a 6‐month mortality rate. Clinical outcome also included a 6‐month unfavorable outcome.

### 2.3 Immunoassay methods

The blood samples of the patients at admission and the controls at study entry were immediately placed on ice and centrifuged at 3000 g. Obtained plasma was aliquoted and frozen at −70°C until assayed. Concentrations of Gal‐3 were in duplicate quantified in plasma by a commercially available kit (BG Medicine, Inc., Waltham, MA, USA) according to the manufacturer's instructions. The person performing the assays was completely blinded to the clinical information.

### 2.4 Statistical analysis

All of statistical analyses were carried out using SPSS 19.0 (SPSS Inc., Chicago, IL, USA) and MedCalc 9.6.4.0 (MedCalc Software, Mariakerke, Belgium). Kolmogorov–Smirnov test was carried out to investigate data distribution. Because WFNS scores and modified Fisher scores were not normally distributed, they were reported as median (interquartile range). Accordingly, the other continuous variables were presented as mean ± standard, and the categorical variables were reported as the number (%). Intergroup differences of continuous data were assessed using *t*‐test. A bivariate correlation analysis was conducted using Spearman's correlation coefficient. Binary logistic regression analyses were performed to investigate independent risk factors for 6‐month clinical outcomes and thus odds ratio (OR) and corresponding 95% confidence interval (CI) were estimated. To assess the prognostic value of Gal‐3, areas under the curve (AUC) of receiver operating characteristics (ROC) curves were calculated. Optimal cut‐off points were determined using ROC curves. All tests were 2‐sided and a *p* value <.05 was considered statistically significant.

## 3 Results

### 3.1 Subject characteristics

**Figure 1:**
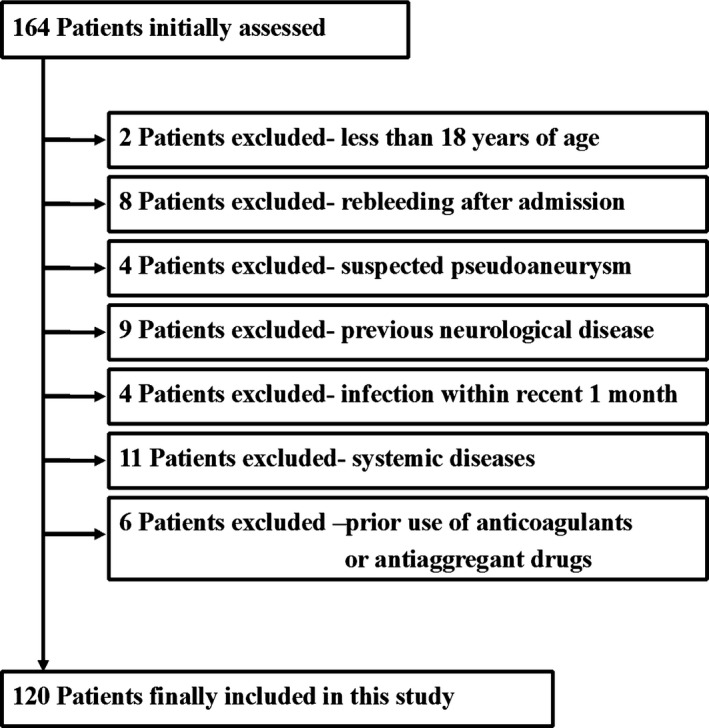
A Flow chart showing the included and excluded patients with aneurysmal subarachnoid hemorrhage in this study

This study initially recruited 164 aSAH patients, and 44 cases were excluded because of the reasons in Fig. 1. A total of 120 aSAH patients were finally included and 120 healthy individuals were recruited as the control group, which was composed of 52 males and 68 females and had a mean age of 48.7 ± 12.4 years. This group of patients had a high percentage of females (50 males and 70 females) and had a mean age of 49.9 ± 13.5 years. There were no significant differences between the two groups in terms of age and gender. The mean WFNS score was 3 (1) and mean modified Fisher score was 3 (1). Thirty‐two (26.7%) aneurysms were located at posterior communication artery; 23 (19.2%), internal carotid artery; 29 (24.2%), anterior communication artery; 20 (16.7%), middle cerebral artery; 9 (7.5%), anterior cerebral artery; 6 (5.0%), posterior cerebral artery; 1 (0.8%), vertebral artery. 56 (46.7%) patients underwent clipping of aneurysms; 64 (53.3%), endovascular coiling of aneurysms. Here 25 (20.9%) patients were complicated by acute hydrocephalus, 16 (13.3%), intraventricular hemorrhage; 19 (15.8%), computed tomography‐confirmed cerebral infarction; 34 (28.3%), symptomatic cerebral vasospasm. The mean admission time was 9.5 ± 4.7 hr and the mean plasma‐sampling time, 12.4 ± 5.3 hr. The patients had a mean systolic arterial pressure of 144.8 ± 24.8 mmHg and a mean diastolic arterial pressure of 87.7 ± 13.7 mmHg. 16.2 ± 5.8 mmol L^−1^ at the mean blood glucose levels and 14.9 ± 4.5 mg L^−1^ at the mean plasma C‐reactive protein levels were found at admission.

### 3.2 Gal‐3 levels analysis

There was significant difference between the patients and controls in terms of plasma Gal‐3 levels (21.7 ± 9.2 ng ml^−1^ vs. 5.2 ± 1.8 ng ml^−1^, *p *<* *.001). Also, plasma Gal‐3 levels were significantly higher in survivors than in nonsurvivors within 6 months (31.6 ± 8.8 ng ml^−1^ vs. 19.9 ± 8.2 ng ml^−1^, *p *<* *.001) and in patients with unfavorable outcome than in those with favorable outcome within 6 months (29.6 ± 8.1 ng ml^−1^ vs. 18.2 ± 7.4 ng ml^−1^, *p *<* *.001). With increasing severity, plasma Gal‐3 levels were significantly elevated. Plasma Gal‐3 levels were markedly associated positively with WFNS scores (*r *= .614, *p *<* *.001) and modified Fisher scores (*r *= .555, *p *<* *.001).

### 3.3 Prognostic prediction analysis

Six‐month mortality was 15.0% (18/120) patients and 37 (30.8%) patients had an unfavorable outcome within 6 months after aSAH. Using univariate binary logistic regression analyses, we found that WFNS scores, modified Fisher scores, Gal‐3 levels and other variables in Tables 1 and 2 were associated significantly with 6‐month mortality and unfavorable outcome. When those variables verified significant in the univariate analyses were further forced into multivariate models, WFNS scores (OR, 5.057; 95% CI, 2.554–15.419; *p *=* *.001), Fisher scores (OR, 3.730; 95% CI, 1.396–9.968; *p *=* *.009), and plasma Gal‐3 levels (OR, 1.129; 95% CI, 1.039–1.227; *p *=* *.004) emerged as the independent predictors for 6‐month mortality; in addition, WFNS scores (OR, 6.800; 95% CI, 2.569–17.997; *p *=* *.001), Fisher scores (OR, 5.957; 95% CI, 2.458–14.434; *p *=* *.004) and plasma Gal‐3 levels (OR, 1.156; 95% CI, 1.049–1.273; *p *=* *.003) were identified as the independent predictors for a 6‐month unfavorable outcome

**Table 1:** The features associated with 6‐month mortality of patients with aneurysmal subarachnoid hemorrhage using a univariate logistic‐regression analyses

	Odds ratio (95% CI)	*p* value
Female	0.672 (0.246–1.836)	.439
Age (year)	1.073 (1.024–1.124)	.003
WFNS scores at admission	7.345 (2.949–19.298)	<.001
Fisher scores at admission	6.605 (2.771–15.741)	<.001
Aneurysmal location	1.076 (0.777–1.489)	.661
Treatment (clipping/coiling)	0.520 (0.181–1.492)	.224
Acute hydrocephalus	4.000 (1.378–11.610)	.011
Intraventricular hemorrhage	4.600 (1.417–14.931)	.011
Computed tomography ischemia	4.773 (1.553–14.670)	.006
Symptomatic cerebral vasospasm	5.398 (1.879–15.506)	.002
Admission time (hr)	0.954 (0.851–1.069)	.415
Plasma‐sampling time (hr)	0.907 (0.812–1.014)	.085
Systolic arterial pressure (mmHg)	1.020 (1.000–1.042)	.054
Diastolic arterial pressure (mmHg)	1.029 (0.990–1.071)	.147
Blood glucose level (mmol L^−1^)	1.159 (1.042–1.289)	.007
Plasma C‐reactive protein level (mg L^−1^)	1.184 (1.055–1.328)	.004
Plasma galectin‐3 level (ng ml^−1^)	1.169 (1.087–1.258)	<.001

95% CI, 95% confidence interval; WFNS, World Federation of Neurological Surgeons.

**Table 2:** The features associated with 6‐month unfavorable outcome of patients with aneurysmal subarachnoid hemorrhage, using univariate logistic‐regression analyses

	Odds ratio (95% CI)	*p* value
Female	0.911 (0.416–1.994)	.815
Age (year)	1.033 (1.002–1.066)	.037
WFNS scores at admission	9.108 (3.963–20.935)	<.001
Fisher scores at admission	9.152 (4.243–19.741)	<.001
Aneurysmal location	1.133 (0.878–1.463)	.337
Treatment (clipping/coiling)	0.594 (0.269–1.311)	.197
Acute hydrocephalus	3.205 (1.289–7.969)	.012
Intraventricular hemorrhage	6.600 (2.097–20.772)	.001
Computed tomography ischemia	5.211 (1.850–14.684)	.002
Symptomatic cerebral vasospasm	3.967 (1.705–9.230)	.001
Admission time (hr)	0.950 (0.871–1.037)	.251
Plasma‐sampling time (hr)	0.969 (0.899–1.045)	.419
Systolic arterial pressure (mmHg)	1.005 (0.989–1.021)	.542
Diastolic arterial pressure (mmHg)	0.992 (0.964–1.021)	.576
Blood glucose level (mmol L^−1^)	1.108 (1.026–1.196)	.009
Plasma C‐reactive protein level (mg L^−1^)	1.120 (1.025–1.223)	.012
Plasma galectin‐3 level (ng ml^−1^)	1.204 (1.120–1.293)	<.001

95% CI, 95% confidence interval; WFNS, World Federation of Neurological Surgeons.

**Figure 2:**
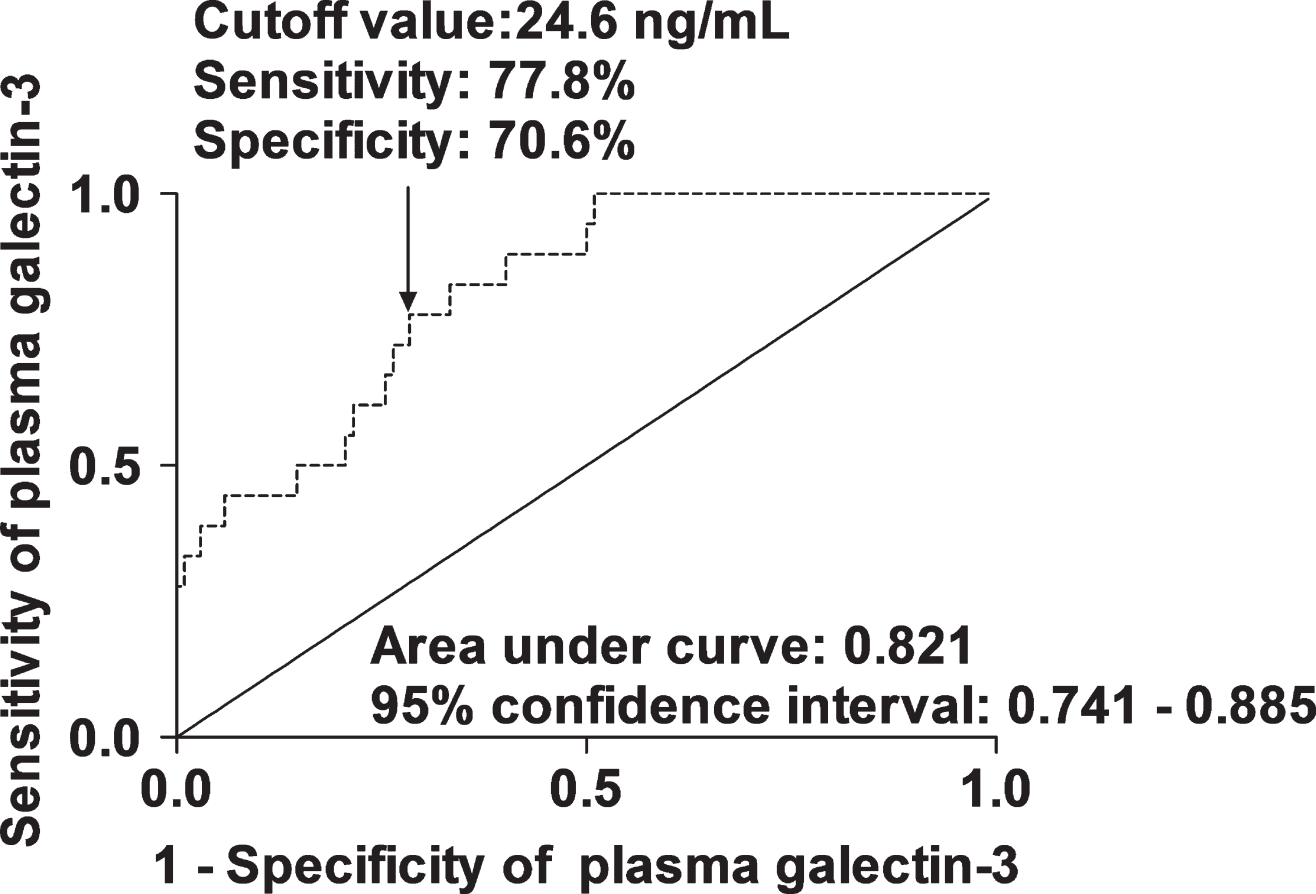
Area under the receiver operating characteristic curve for identifying aneurysmal subarachnoid hemorrhage patients at risk of 6‐month mortality based on plasma galectin‐3 levels

**Figure 3:**
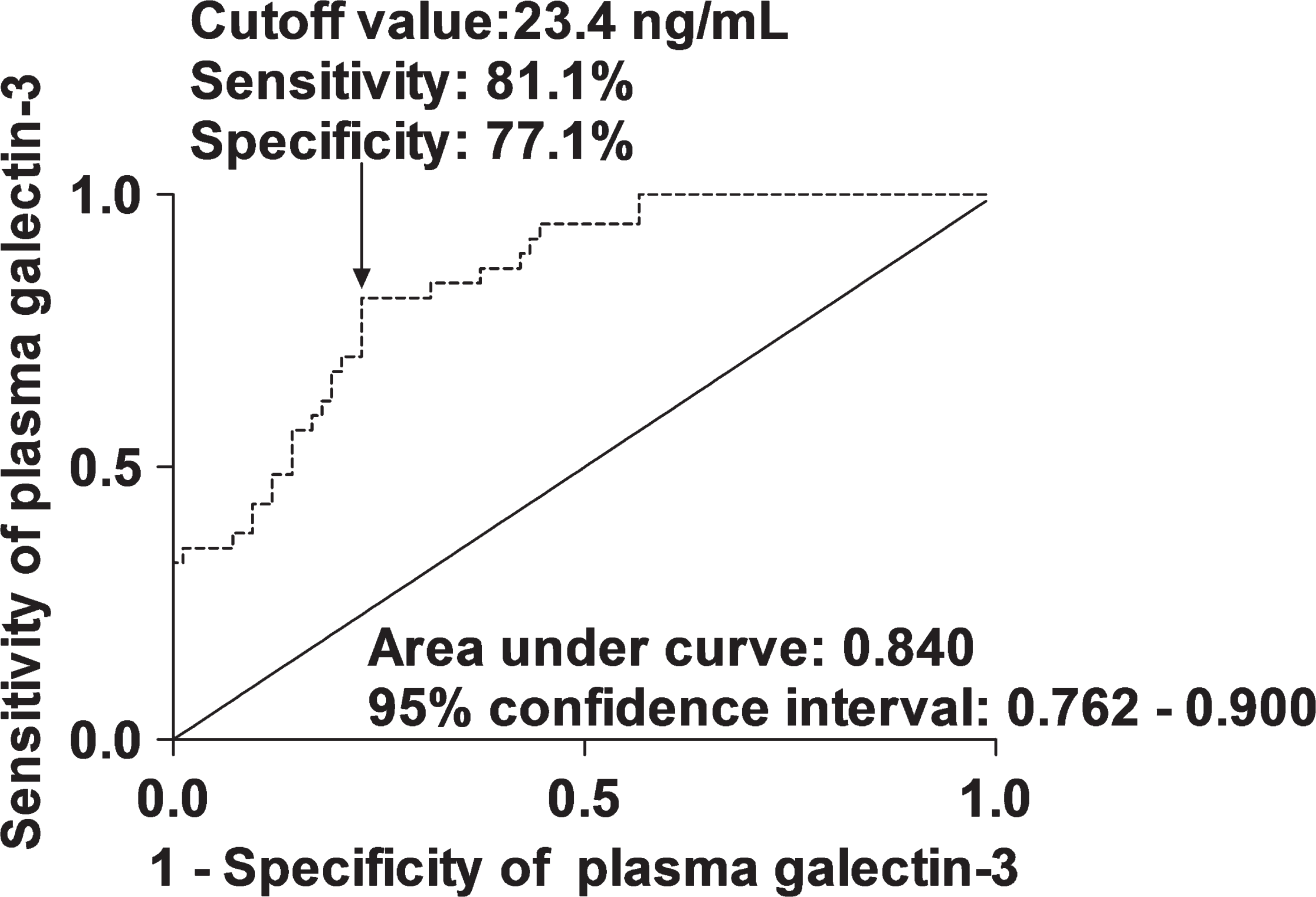
Area under the receiver operating characteristic curve for identifying aneurysmal subarachnoid hemorrhage patients at risk of 6‐month unfavorable outcome based on plasma galectin‐3 levels

Using ROC curve analyses, Figs 2, 3 show that plasma Gal‐3 levels statistically significantly predicted a 6‐month mortality and an unfavorable outcome. Moreover, in Table 3, plasma Gal‐3 levels had similar predictive value with WFNS scores and Fisher scores; however, using combined logistic‐regression models, Gal‐3 levels numerically improved AUCs of WFNS scores and Fisher scores for prediction of a 6‐month mortality and an unfavorable outcome.

**Table 3:** The receiver operating characteristic curve analysis for 6‐month poor prognosis

Variables	AUC (95% CI)	*p* value	*p* value
A 6‐month mortality			
WFNS scores	0.870 (0.796–0.924)	Ref.	
Fisher scores	0.846 (0.769–0.905)		Ref.
Plasma galectin‐3 levels	0.821 (0.741–0.885)	.475	.675
WFNS scores combined with plasma galectin‐3 levels	0.922 (0.858–0.963)	.187	
Fisher scores combined with plasma galectin‐3 levels	0.877 (0.804–0.930)		.343
A 6‐month unfavorable outcome			
WFNS scores	0.885 (0.814–0.936)	Ref.	
Fisher scores	0.876 (0.804–0.929)		Ref.
Plasma galectin‐3 levels	0.840 (0.762–0.900)	.371	.458
WFNS scores combined with plasma galectin‐3 levels	0.921 (0.857–0.962)	.161	
Fisher scores combined with plasma galectin‐3 levels	0.913 (0.848–0.957)		.163

AUC, area under curve; WFNS, World Federation of Neurological Surgeons; Ref., reference; 95% CI, confidence interval.

## 4 Discussion

The main findings from this prospective, observatory study on the change of Gal‐3 levels in aSAH were as follows: at first, plasma Gal‐3 levels were significantly enhanced after aSAH; secondly, plasma Gal‐3 levels were related closely to the admission WFNS scores and modified Fisher scores; thirdly, Gal‐3 was an independent predictor for 6‐month mortality and unfavorable outcome; Finally, based on AUC, the predictive value of plasma Gal‐3 levels resembled those of WFNS scores and modified Fisher scores.

Since a recent paper reported that plasma Gal‐3 concentrations were elevated after severe traumatic brain injury (Shen et al., 2016), to our best knowledge, there had been a paucity of the data available on change of plasma Gal‐3 levels following aSAH. It is the first study to measure circulating Gal‐3 levels in such a group of patients with aSAH. Here, we reported the significantly elevated Gal‐3 levels in plasma of aSAH patients. Although Gal‐3 is widely distributed in both species and tissues (Almkvist & Karlsson, 2004; Arar, Gaudin, Capron, & Legrand, 1998; Yang, Rabinovich, & Liu, 2008), it is also derived from glia (Jaquenod De Giusti et al., 2011) and its expression is markedly up‐regulated in response to some insult such as ischemia (Lalancette‐Hébert et al., 2012). Moreover, this biomarker was found to be elevated in cerebrospinal fluid from newborn infants after birth asphyxia (Sävman, Heyes, Svedin, & Karlsson, 2013). Together, these finding indicate that Gal‐3 might be derived from brain tissues after hemorrhagic injury.

The actual roles of Gal‐3 in central nervous systemic tissues remain unknown. However, the accumulating evidence has shown that Gal‐3 might be essential for normal brain development where it plays diverse biological roles, including cell‐to‐cell adhesion and cell migration (Pesheva, Kuklinski, Biersack, & Probstmeier, 2000; Pesheva, Kuklinski, Schmitz, & Probstmeier, 1998). In various types of brain inflammatory disorders, including ischemic brain injury, experimental autoimmune encephalomyelitis, and virus‐induced encephalitis, Gal‐3 is presumed to have pleiotropic effects, including a proinflammatory role and a remodeling capacity in injured brain tissues (Doverhag et al., 2010; Jaquenod De Giusti et al., 2011; Jiang et al., 2009). In keeping with the results on the above‐mentioned diseases with acute brain injury (Doverhag et al., 2010; Jaquenod De Giusti et al., 2011; Jiang et al., 2009), further study is warranted to address the potential effects of Gal‐3 in hemorrhagic brain injury after aSAH

A recent study had indicated that elevated plasma Gal‐3 levels were highly associated with clinical severity reflected by Glasgow coma scale and Gal‐3 independently predicted in‐hospital mortality and identified patients as a great risk of adverse complications, including acute traumatic coagulopathy, progressive hemorrhagic injury, and posttraumatic cerebral infarction (Shen et al., 2016). Similarly, our current study demonstrated that Gal‐3 levels were related closely to hemorrhagic severity indicated by WFNS scores and modified Fisher scores. Moreover, we extended the clinical follow‐up interval to 6 months and assess the neurological function outcome, and found interesting results that elevated plasma Gal‐3 levels had a close relation to a 6‐month mortality and an unfavorable outcome. In line with previous data in patients with severe traumatic brain that Gal‐3 levels had high predictive value for in‐hospital mortality and adverse complications based on ROC curve analysis (Shen et al., 2016), our study confirmed that Gal‐3 levels also possessed high prognostic value for 6‐month clinical outcomes and its AUC was similar to those of WFNS scores and modified Fisher scores. Actually, our findings extend the previous study (Shen et al., 2016) and substantialize Gal‐3 as a potential prognostic biomarker. Overall, circulating Gal‐3 levels might be relevant to the severity and prognosis following aSAH.

## 5 Conclusions

This study demonstrates that plasma Gal‐3 levels are enhanced after aSAH and correlated positively with the WFNS scores and modified Fisher scores. Gal‐3 levels independently predict 6‐month mortality and unfavorable outcome with a high predictive value. Thus, Gal‐3 may aid in predicting the risk of poor prognosis in patients with aSAH.

## Conflict of Interest

The authors have no conflict of interest.
